# Genetic Risk, Lifestyle, and Colorectal Cancer Across Age at Onset: A Prospective UK Biobank Study

**DOI:** 10.1002/cam4.72451

**Published:** 2026-10-29

**Authors:** Wen Sun, Di Liu, Jingyang Liu, Dongxue Wang, Xiao Wang, Deqiang Zheng, Jianguang Ji

**Affiliations:** ^1^ Department of Epidemiology and Health Statistics, School of Public Health Capital Medical University Beijing China; ^2^ Beijing Key Laboratory of Environment and Aging Capital Medical University Beijing China; ^3^ Faculty of Medicine University of Macau Macau SAR China; ^4^ Center for Primary Health Care Research, Department of Clinical Sciences Malmö Lund University Malmö Sweden

**Keywords:** colorectal cancer, early‐onset, late‐onset, lifestyle, polygenic risk score

## Abstract

**Background:**

Polygenic risk scores (PRS) and lifestyle factors are associated with colorectal cancer (CRC), but whether their associations and joint effects vary according to age at CRC onset remains unclear. We evaluated age‐related heterogeneity in the associations of genetic risk and lifestyle with CRC and examined their additive interaction in early‐ and late‐onset CRC.

**Methods:**

We analyzed data from 405,453 cancer‐free participants aged 38–73 years at baseline in the UK Biobank. PRS and a healthy lifestyle score (based on eight modifiable factors) were assessed. Age‐scale Cox proportional hazards models estimated hazard ratios (HRs) for CRC risk by age at onset. Heterogeneity across age intervals was assessed using likelihood ratio tests. Additive interaction was assessed using the relative excess risk due to interaction (RERI), and differences in standardized incidence rates were evaluated.

**Results:**

During a median follow‐up of 13.65 years, 6620 CRC cases were identified. Compared with low genetic risk, high genetic risk was associated with HRs of 3.72, 3.32, 2.80, and 2.01 for CRC occurring at ages < 55, 55–64, 65–74, and ≥ 75 years, respectively, with significant heterogeneity across age intervals (*p* for heterogeneity < 0.001). Compared with a favorable lifestyle, the corresponding HRs for an unfavorable lifestyle were 1.10, 1.50, 1.58, and 1.28, respectively, with evidence of overall heterogeneity (*p* for heterogeneity = 0.015). For late‐onset CRC, positive additive interactions were observed for an unfavorable lifestyle combined with intermediate or high genetic risk, whereas no such interaction was detected for early‐onset CRC.

**Conclusions:**

The associations of genetic susceptibility and baseline lifestyle with CRC varied according to age at onset. Positive additive interaction between genetic risk and an unfavorable lifestyle was observed for late‐onset but not early‐onset CRC. These findings provide further evidence that the relative and joint associations of genetic and lifestyle factors with CRC vary across the life course.

## Graphical abstract

Association of genetic risk and lifestyle with early‐ and late‐onset colorectal cancer in the UK Biobank. Genetic risk was more strongly associated with early‐onset colorectal cancer, whereas significant additive interactions between genetic risk and lifestyle were observed for late‐onset colorectal cancer.

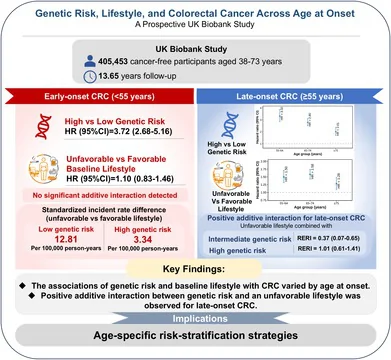

## 1 Introduction

Colorectal cancer (CRC) incidence has generally stabilized or declined in high‐income countries, largely due to increased screening rates, widespread adoption of colonoscopic polypectomy, and improvements in lifestyles [1]. In contrast, the global incidence of early‐onset CRC has risen sharply since the early 1990s [1, 2, 3]. In the United States, early‐onset CRC has become the leading cause of cancer mortality among young men and the second leading cause among young women [4]. Early‐onset CRC accounts for about 10% of all CRC cases [5], and this proportion is projected to rise to one‐third by 2030 [6]. Compared with late‐onset CRC, early‐onset CRC exhibits distinct clinical characteristics and is more frequently diagnosed at an advanced stage [7]. This alarming trend has drawn increasing attention in recent years, prompting many studies to investigate the underlying risk factors associated with its development [8, 9, 10, 11].

Both inherited susceptibility and modifiable exposures contribute to CRC risk [12]. Genome‐wide association studies (GWAS) have identified numerous CRC‐associated variants [13, 14, 15], which have been aggregated into polygenic risk scores (PRS) to capture inherited susceptibility [10, 16, 17, 18]. Several studies have suggested that genetic susceptibility may differ according to age at CRC onset. Archambault et al. [19] reported a stronger cumulative burden of CRC‐associated genetic variants in early‐onset than late‐onset CRC, and subsequent studies using the UK Biobank and other populations have also demonstrated important associations between PRS and CRC diagnosed at younger ages [10, 20, 21, 22]. Accumulating evidence indicates that lifestyle and other modifiable factors may also contribute differently to CRC according to age at onset. Previous studies have compared anthropometric, dietary, metabolic, smoking, alcohol, and other environmental factors between early‐ and late‐onset CRC [11, 23, 24, 25, 26].

Furthermore, several studies have evaluated multiplicative or additive interactions between PRS and lifestyle‐related exposures, including comprehensive healthy lifestyle scores, dietary patterns, alcohol consumption, and tobacco exposure. Archambault et al. [27] combined polygenic and environmental risk scores for early‐onset CRC risk stratification, and some studies also demonstrated the value of considering genetic and environmental factors jointly [12, 28, 29, 30, 31, 32, 33].

Despite these advances, several questions remain unresolved. First, it is not well established whether the associations of polygenic susceptibility and a comprehensive lifestyle profile with CRC change systematically across multiple ages of onset rather than simply between early‐ and late‐onset diseases. Second, the continuous age‐related variation in these associations has not been well characterized within a prospective time‐to‐event framework. Third, although previous studies have examined joint or interactive effects of genetic and environmental factors, whether additive interaction between genetic susceptibility and a comprehensive lifestyle profile differs according to CRC age at onset remains unclear. Clarifying these age‐specific patterns is important for understanding how the associations of genetic susceptibility and lifestyle vary across age at CRC onset and may inform future age‐specific risk stratification and prevention research.

To address these gaps, we used a large prospective cohort from the UK Biobank to evaluate the associations of CRC PRS and an eight‐component healthy lifestyle score with incident CRC across four onset‐age intervals (< 55, 55–64, 65–74, and ≥ 75 years). We formally tested heterogeneity in these associations across age intervals and further characterized their continuous variation with onset age. In addition, we examined additive interactions between genetic risk and lifestyle separately for early‐ and late‐onset CRC. By integrating multiple onset‐age intervals, continuous age‐varying associations, and additive interaction within the same prospective framework, this study extends previous work on the age‐dependent contributions of genetic susceptibility and lifestyle to CRC.

## 2 Methods

### 2.1 Study Participants

Participants were selected from the UK Biobank, a population‐based prospective cohort with baseline phenotypic and genetic information. The baseline assessment included genotype data, physical measurements, lifestyle information, personal medical history, and family history. Subsequent health events were ascertained through linked cancer, hospital, primary care, and mortality records.

We excluded individuals who were outliers for heterozygosity, had sex chromosome aneuploidy or sex mismatches, had 10 or more third‐degree relatives, had a history of cancer at baseline, or had missing data on PRS, lifestyle factors, or covariates. Early‐onset CRC cases were defined as diagnosis before age 55, considering that participants in the UK Biobank tend to be healthier than the general population [34] and to ensure an adequate number of cases in the younger population for our analyses [23] (Figure S1). Finally, 405,453 participants aged 38–73 years were included in the primary analysis.

### 2.2 Genomic Data and PRS Construction

We used the standard CRC PRS released by the UK Biobank. Details of genotyping, imputation, quality control, and PRS development have been reported elsewhere [35, 36]. Briefly, the CRC PRS was developed using summary statistics from four external genome‐wide association studies that did not include UK Biobank participants. For each participant, the score was calculated as the genome‐wide sum of allele dosages weighted by their corresponding effect estimates. Participants were classified into three genetic‐risk categories according to the PRS distribution: low risk (bottom quartile), intermediate risk (second and third quartiles), and high risk (top quartile).

### 2.3 Assessment of Lifestyles

A healthy lifestyle score was derived from eight baseline characteristics [12], including body mass index (BMI), smoking, alcohol consumption, waist‐to‐hip ratio (WHR), physical activity, sedentary time, red and processed meat intake, and vegetable and fruit intake (Table S1). Information on these lifestyle factors was collected at baseline through standardized touchscreen questionnaires and physical measurements. Participants completed self‐administered questionnaires that assessed smoking status, alcohol intake, physical activity, sedentary behaviors, and dietary habits. BMI and WHR were calculated from physical measurements. BMI was calculated from measured height and weight, and WHR was derived from waist and hip circumference measurements. Each component contributed one point when the prespecified healthy criterion was met. The resulting score ranged from 0 to 8 and was grouped as unfavorable (0–1), intermediate (2–3), or favorable (≥ 4).

### 2.4 Definition of Outcomes

Incident CRC was defined by the International Classification of Diseases (ICD‐10) codes C18–C20 and identified through linked cancer registry, hospital, and primary care records. The first recorded CRC diagnosis during follow‐up was used as the study outcome.

### 2.5 Statistical Analysis

Baseline characteristics were summarized according to subsequent CRC status and genetic‐risk category. Associations of genetic risk and baseline lifestyle with CRC were estimated using Cox proportional hazards regression. Age was used as the underlying timescale, and follow‐up time was censored at the age of CRC onset, death, loss to follow‐up, or the last censoring date (October 31, 2022, for England; August 31, 2022, for Scotland; and May 31, 2022, for Wales), whichever came first. All models were additionally adjusted for sex (men or women), ethnicity (White or other), Townsend deprivation index, and family history of CRC (yes or no).

Tests based on Schoenfeld residuals indicated that the proportional hazards assumption was not satisfied for genetic‐risk or lifestyle categories over the entire follow‐up period. We therefore allowed the corresponding regression coefficients to vary across four onset‐age intervals: < 55, 55–64, 65–74, and ≥ 75 years. Heterogeneity in the associations of genetic risk and lifestyle with CRC across onset‐age intervals was assessed using likelihood ratio tests. We compared Cox models that allowed the exposure coefficient to vary across age intervals with corresponding models that assumed a common exposure coefficient across all intervals [26, 37, 38]. Additional category‐specific and pairwise comparisons were performed. For pairwise analyses, the < 55‐year interval was used as the reference. Hazard ratios (HRs) and 95% confidence intervals (CIs) were also calculated per 1‐standard deviation (SD) increase in the PRS and per 1‐unit decrease in the healthy lifestyle score. To further allow coefficients to vary continuously over time, we used splines by constructing interaction terms between onset age during follow‐up and PRSs or lifestyle scores [39, 40]. In addition, we examined the associations of each lifestyle factor with early‐ and late‐onset CRC risk within different genetic risk groups. The cumulative incidence function (CIF) for CRC in each PRS or lifestyle group was calculated, which accounts for the competing risk of non‐CRC death [41].

Additive interaction was assessed using the relative excess risk due to interaction (RERI). We further partitioned the joint association of genetic risk and unfavorable lifestyle into contributions from genetic risk alone, lifestyle alone, and their interaction, and summarized these components using the alternative attributable proportion (AAP) [40, 42, 43]. Standardized incidence rates of early‐ and late‐onset CRC were estimated for combinations of genetic risk and baseline lifestyle categories using the mean distribution of the model covariates. Differences in standardized incidence rates between lifestyle categories were used to describe absolute contrasts in CRC occurrence. CIs for interaction measures and standardized incidence rate differences were obtained from 1000 bootstrap resamples.

Several sensitivity analyses were performed to assess the robustness of our findings. First, we repeated the analyses after excluding 140 early‐onset CRC cases diagnosed within 6 months of an endoscopic examination to reduce the possibility of opportunity diagnosis related to recent endoscopy. Second, we defined early‐onset CRC as a diagnosis before age 50 and alternatively divided the time axis into four age groups (< 50, 50–59, 60–69, and ≥ 70 years) to test the stability of the age stratification. Third, we excluded events that occurred within the first year of follow‐up to minimize potential reverse causation.

All tests were two‐sided and used a significance threshold of *p* < 0.05. Analyses were conducted in R version 4.4.3.

## 3 Results

### 3.1 Population Characteristics

A total of 405,453 participants aged 38–73 years were included in the analysis. The baseline characteristics are presented in Table 1 and Tables S2 and S3. The median age was 57 (IQR, 49–63) years. During a median follow‐up of 13.65 (IQR, 12.92–14.28) years (5,377,191 person‐years), there were 6620 cases of incident CRC with an incidence rate of 1.23 per 1000 person‐years in the total population. Of these cases, 387 were diagnosed before age 55 years (204 women, 183 men) and 6233 were diagnosed at age 55 years or older (2702 women and 3531 men) (Table 1). Compared with participants at low genetic risk, those with intermediate and high genetic risk were more likely to be male, White, have a family history of CRC, have an unfavorable lifestyle, and exhibit a higher incidence of both early‐ and late‐onset CRC (Table S2). Participants who developed early‐onset CRC, compared with those with late‐onset CRC, were more likely to be female, non‐White, and have a higher proportion of high genetic risk (Table 1).

**TABLE 1:** Characteristics of participants at baseline according to non‐, early‐ and late‐onset colorectal cancer (CRC).

Characteristics	No incident CRC (*N* = 398,833)	Incident CRC	
		Early‐onset^a^ (*N* = 387)	Late‐onset^b^ (*N* = 6233)
Age at baseline, median (IQR)	57 (49–63)	45 (43–49)	62 (57–66)
Follow‐up time, median (IQR)	13.67 (12.95–14.29)	4.15 (2.18–7.67)	8.04 (4.39–11.06)
Person‐years^c^	5,327,475	1901	47,816
Sex, *N* (%)			
Female	211,871 (53.12)	204 (52.71)	2702 (43.35)
Male	186,962 (46.88)	183 (47.29)	3531 (56.65)
Ethnicity, *N* (%)			
White	378,835 (94.99)	363 (93.80)	6075 (97.47)
Other	19,998 (5.01)	24 (6.20)	158 (2.53)
Townsend deprivation index, mean (SD)	−1.39 (3.04)	−0.96 (3.19)	−1.54 (3.01)
Genetic risk groups^d^, *N* (%)			
Low	100,439 (25.18)	45 (11.63)	880 (14.12)
Intermediate	199,613 (50.05)	172 (44.44)	2941 (47.18)
High	98,781 (24.77)	170 (43.93)	2412 (38.70)
Lifestyle scores^e^, *N* (%)			
Favorable	135,533 (33.98)	136 (35.14)	1564 (25.09)
Intermediate	180,154 (45.17)	176 (45.48)	2807 (45.03)
Unfavorable	83,146 (20.85)	75 (19.38)	1862 (29.87)
Family history of CRC, *N* (%)			
Yes	44,320 (11.11)	57 (14.73)	944 (15.15)
No	354,513 (88.89)	330 (85.27)	5289 (84.85)

Abbreviations: CRC, colorectal cancer; IQR, interquartile range; PRS, polygenic risk score; SD, standard deviation.

^a^ Early‐onset CRC was defined as diagnosis before age 55.

^b^ Late‐onset CRC was defined as diagnosis at age 55 or older.

^c^ Person‐years were calculated as the sum of individual follow‐up times within each group. Follow‐up time was calculated from the date of baseline assessment to the date of CRC diagnosis, death, loss to follow‐up, or the last censoring date.

^d^ Genetic risk was categorized into low (bottom quartile), intermediate (2nd–3rd quartile) and high (top quartile) according to quartile cutoff points of the PRS.

^e^ Participants were classified into unfavorable (0 and 1 score), intermediate (2 and 3 score), and favorable (≥ 4 score) lifestyle subgroups based on lifestyle scores ranging from 0 to 8.

### 3.2 Genetic Risk, Lifestyles and Different Age Onsets of CRC

The cumulative incidence of CRC ranged from 2.61% to 6.72% across increasing PRS categories. Similarly, cumulative incidence ranged from 3.72% among individuals with a favorable lifestyle to 5.40% among those with an unfavorable lifestyle (Table S4 and Figure S2).

Compared with participants at low genetic risk, those at high genetic risk exhibited HRs (95% CIs) for CRC of 3.72 (2.68–5.16) before age 55 years, 3.32 (2.85–3.88) at ages 55–64 years, 2.80 (2.52–3.11) at ages 65–74 years, and 2.01 (1.71–2.38) at ages 75 years or older, respectively (*p* for heterogeneity < 0.001) (Table 2). The heterogeneity was particularly evident for high versus low genetic risk (*p* for heterogeneity < 0.001), whereas the evidence was weaker for intermediate versus low genetic risk (*p* for heterogeneity = 0.053) (Table S5). In pairwise comparisons using CRC diagnosed before age 55 years as the reference, the genetic‐risk association did not differ significantly for CRC diagnosed at ages 55–64 years (*p* for heterogeneity = 0.750) or 65–74 years (*p* for heterogeneity = 0.142), but differed for CRC onset at age ≥ 75 years (*p* for heterogeneity = 0.002) (Table S6).

**TABLE 2:** Associations of genetic risk and lifestyles with colorectal cancer (CRC).

Age group of onset	Case	Case per PY (1000)	Genetic risk, HR (95% CI)				Lifestyle, HR (95% CI)			
			Low	Intermediate	High	*p* for heterogeneity	Favorable	Intermediate	Unfavorable	*p* for heterogeneity
< 55	387	0.32	Reference	1.90 (1.37–2.64)	3.72 (2.68–5.16)	< 0.001	Reference	1.07 (0.86–1.34)	1.10 (0.83–1.46)	0.015
55–64	1695	0.89	Reference	1.83 (1.57–2.13)	3.32 (2.85–3.88)		Reference	1.25 (1.11–1.40)	1.50 (1.32–1.71)	
65–74	3317	1.68	Reference	1.72 (1.55–1.91)	2.80 (2.52–3.11)		Reference	1.24 (1.13–1.35)	1.58 (1.43–1.74)	
≥ 75	1221	2.53	Reference	1.38 (1.18–1.62)	2.01 (1.71–2.38)		Reference	0.96 (0.83–1.10)	1.28 (1.10–1.48)	

*Note:* Adjusted for sex, ethnicity, Townsend deprivation index, and family history of CRC.

Abbreviations: CI, confidence interval; CRC, colorectal cancer; HR, hazard ratio; PY, person‐years.

Relative to a favorable lifestyle, the corresponding HRs (95% CIs) for an unfavorable lifestyle were 1.10 (0.83–1.46), 1.50 (1.32–1.71), 1.58 (1.43–1.74), and 1.28 (1.10–1.48), respectively (*p* for heterogeneity = 0.015) (Table 2). The heterogeneity across onset‐age intervals was observed for both intermediate versus favorable lifestyle (*p* for heterogeneity = 0.010) and unfavorable versus favorable lifestyle (*p* for heterogeneity = 0.019) (Table S5). The heterogeneity was mainly attributable to the unfavorable versus favorable lifestyle contrast, particularly when comparing CRC occurring before age 55 years with CRC occurring at ages 55–64 (*p* for heterogeneity = 0.046) and 65–74 years (*p* for heterogeneity = 0.015) (Table S7).

Similar age‐related heterogeneity was observed when PRS and lifestyle scores were modeled continuously. The HRs per 1‐SD increase in PRS were 1.54, 1.58, 1.47, and 1.34 across the four onset‐age intervals (*p* for heterogeneity < 0.001), whereas the corresponding HRs per one‐factor decrease in the healthy lifestyle score were 1.02, 1.10, 1.12, and 1.07 (*p* for heterogeneity = 0.023) (Table S8). As illustrated in Figure S3, the association between genetic risk and CRC risk declined gradually with increasing onset age, whereas the age‐related pattern for lifestyle was less consistent, highlighting distinct age‐specific effects of genetic and lifestyle factors on CRC development.

### 3.3 Joint Effect of Genetic Risk and Lifestyles on Early‐ and Late‐Onset CRC

Figure 1 shows the associations of lifestyle categories with the risk of incident early‐ and late‐onset CRC across strata of genetic risk. For early‐onset CRC, there was no statistically significant association between an unfavorable versus favorable lifestyle within any genetic‐risk stratum: the HRs (95% CIs) were 1.90 (0.84–4.32), 1.30 (0.84–2.01), and 0.98 (0.61–1.58) in the low, intermediate, and high genetic risk groups, respectively (Figure 1A). For late‐onset CRC, an unfavorable lifestyle was associated with higher CRC risk across all genetic‐risk strata. The HRs (95% CIs) were 1.37 (1.13–1.65) in the low genetic risk group, 1.46 (1.31–1.62) in the intermediate group, and 1.51 (1.34–1.69) in the high genetic risk group (Figure 1B).

**FIGURE 1:**
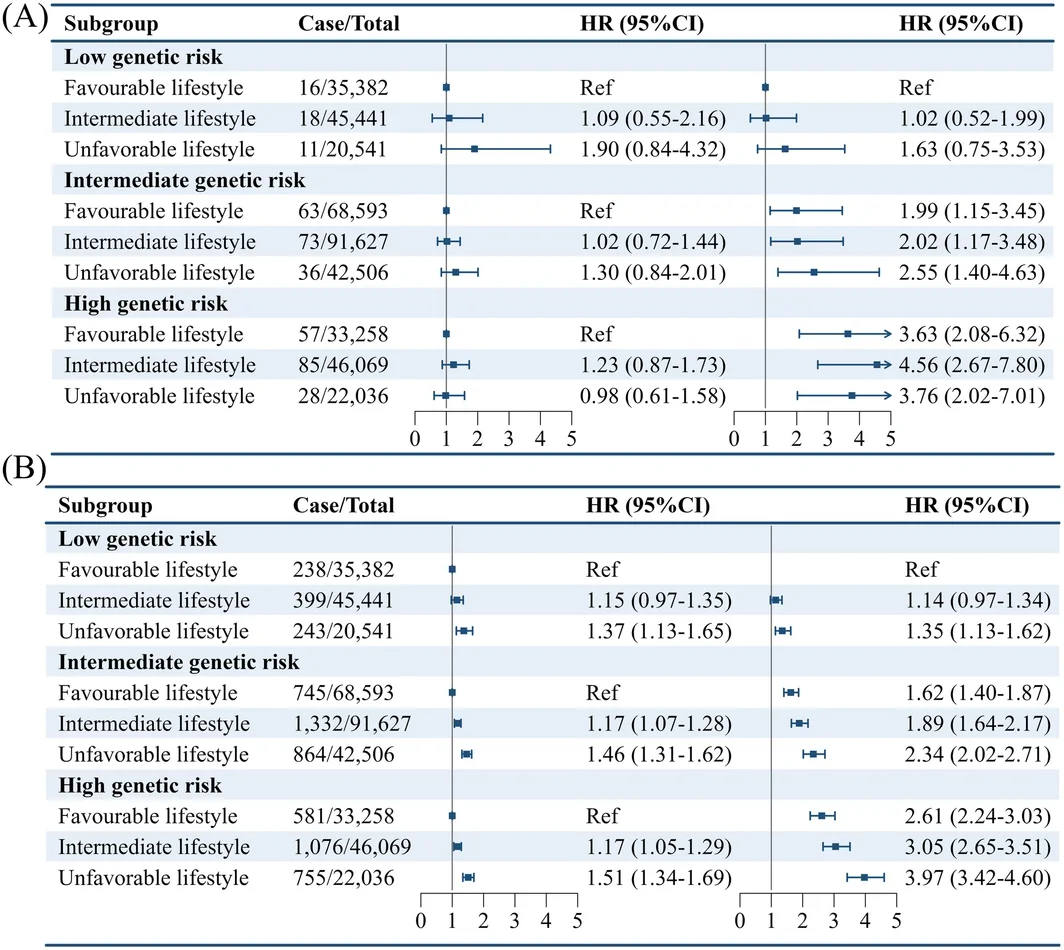
Risk of incident early‐ and late‐onset colorectal cancer (CRC) according to lifestyle category within genetic risk strata. (A) Early‐onset CRC. (B) Late‐onset CRC. Hazard ratios (HRs) and 95% confidence intervals (CIs) were estimated using Cox proportional hazards models adjusted for sex, ethnicity, Townsend deprivation index, and family history of CRC. Statistical tests were two‐sided. The squares indicate the HR estimates, and the horizontal lines represent the corresponding 95% CIs. CI, confidence interval; CRC, colorectal cancer; HR, hazard ratio.

We explored the associations of each lifestyle component with early‐ and late‐onset CRC risk stratified by genetic risk groups (Tables S9 and S10). For early‐onset CRC, high meat intake was associated with a higher risk of early‐onset CRC in the low genetic risk group (HR = 2.09, 95% CI: 1.13–3.86) (Table S9). For late‐onset CRC, smoking and WHR were strongly associated with late‐onset CRC risk (Table S10).

Positive additive interactions between genetic risk and lifestyle were observed for late‐onset CRC but not for early‐onset CRC (Table 3). For late‐onset CRC, the RERIs for an unfavorable lifestyle combined with intermediate and high genetic risk were 0.37 (95% CI, 0.07–0.65) and 1.01 (95% CI, 0.61–1.41), respectively (Table 3). Compared with individuals at low genetic risk and with a favorable lifestyle, individuals with both intermediate or high genetic risk and an unfavorable lifestyle had substantially increased risk of late‐onset CRC (HR = 2.34, 95% CI: 2.02–2.71; HR = 3.97, 95% CI: 3.42–4.60) (Figure 1B). The AAP due to genetic risk alone was 45.91% and 54.09% for intermediate and high genetic risk groups, respectively. The AAP attributable to an unfavorable lifestyle alone was 26.37% and 11.91%. The AAP for the interaction between genetic risk and an unfavorable lifestyle reached 27.72% and 34.00%, indicating a significant positive additive interaction on late‐onset CRC risk (Table 4).

**TABLE 3:** Relative excess risks due to interactions between genetic risk and lifestyles on early‐ and late‐onset colorectal cancer (CRC).

Outcome	Age of onset	Intermediate genetic risk, RERI (95% CI)		High genetic risk, RERI (95% CI)	
		Intermediate lifestyle	Unfavorable lifestyle	Intermediate lifestyle	Unfavorable lifestyle
Early‐onset CRC	< 55	0.01 (−1.34–0.83)	−0.07 (−2.05–1.30)	0.92 (−0.75–2.38)	−0.50 (−3.52–1.51)
Late‐onset CRC	≥ 55	0.13 (−0.13–0.35)	**0.37 (0.07–0.65)**	0.30 (−0.04–0.59)	**1.01 (0.61–1.41)**

*Note:* Adjust for sex, ethnicity, Townsend deprivation index, and family history of CRC. The 95% CIs were derived by drawing 1000 bootstrap samples. Bold values indicate statistically significant additive interactions.

Abbreviations: CI, confidence interval; CRC, colorectal cancer; RERI, relative excess risks due to interaction.

**TABLE 4:** Alternative attributable proportion (%) measures for interaction between genetic risk and unfavorable lifestyle on late‐onset colorectal cancer (CRC).

Component	Intermediate genetic risk & unfavorable lifestyle AAP (95% CI)	High genetic risk & unfavorable lifestyle AAP (95% CI)
Genetic risk only	45.91 (33.83–58.14)	54.09 (45.63–64.00)
Unfavorable lifestyle only	26.37 (11.53–41.87)	11.91 (5.09–19.14)
Interaction	27.72 (4.42–50.83)	34.00 (21.01–45.64)

*Note:* The alternative attributable proportion measures were calculated with low genetic risk and favorable lifestyle as the reference group. Late‐onset CRC was defined as age of onset ≥ 55 years. The 95% CIs were derived by drawing 1000 bootstrap samples.

Abbreviations: AAP, alternative attributable proportion; CI, confidence interval.

The absolute differences in the standardized incidence rates (per 100,000 person‐years) between participants with unfavorable and favorable baseline lifestyles varied across genetic risk strata (Figure 2). For early‐onset CRC, the rate difference was 12.81 cases per 100,000 person‐years in the low genetic risk group (32.88 vs. 20.07) and 3.34 cases per 100,000 person‐years in the high genetic risk group (76.17 vs. 72.83), corresponding to a 3.8‐fold larger absolute difference in the low than in the high genetic risk group (Figure 2A). In contrast, for late‐onset CRC, the corresponding rate difference was 84.87 cases per 100,000 person‐years in the high genetic risk group (247.99 vs. 163.12), compared with 21.91 cases per 100,000 person‐years in the low genetic risk group (84.48 vs. 62.57), representing an approximately 3.9‐fold larger absolute difference in the high than in the low genetic risk group (Figure 2B).

**FIGURE 2:**
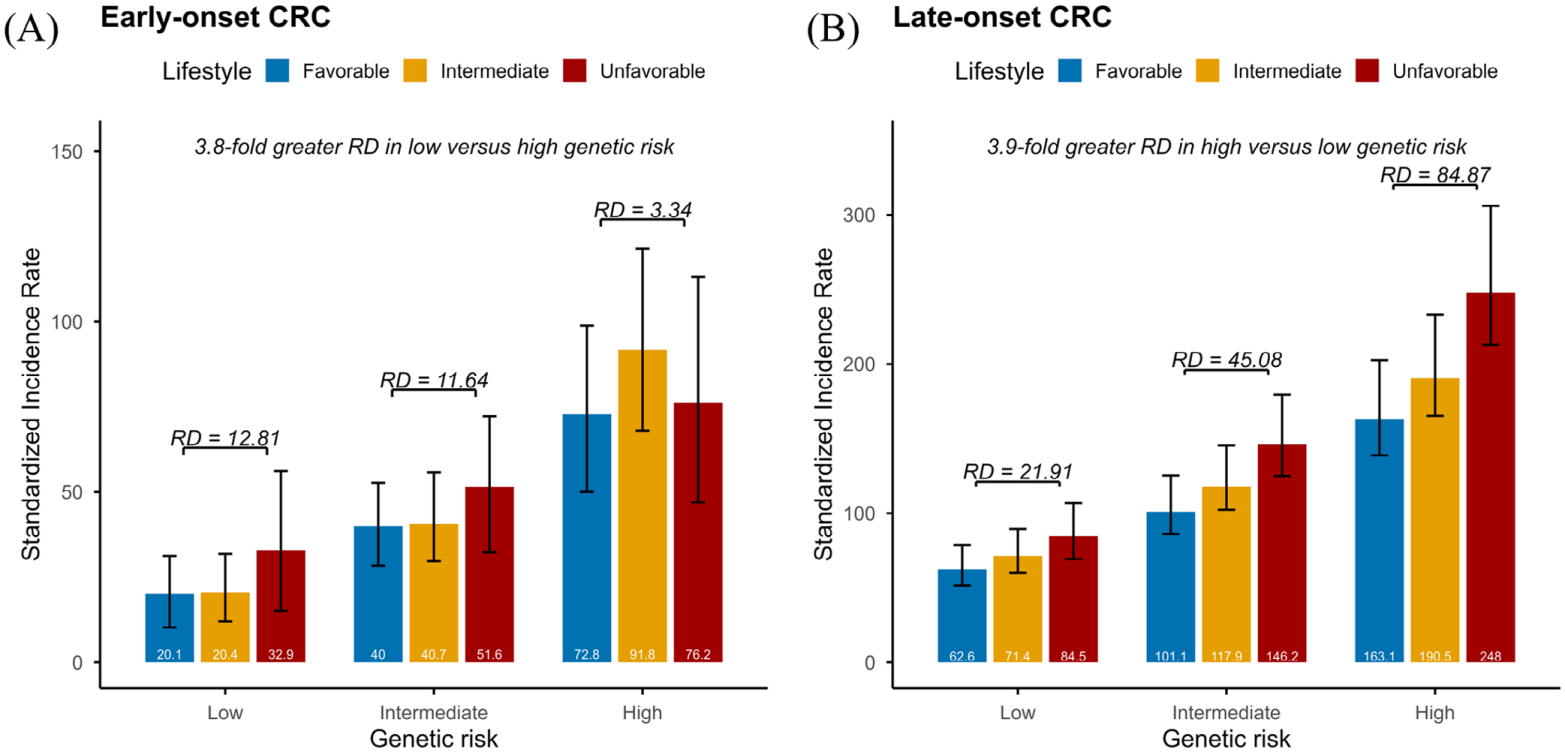
Standardized incidence rates (per 100,000 person‐years) of early‐ and late‐onset colorectal cancer (CRC) according to genetic risk and lifestyle. (A) Early‐onset CRC. (B) Late‐onset CRC. The 95% CIs were derived by drawing 1000 bootstrap samples. The error bars represent 95% CIs and their centers represent the standardized incidence rates. CI, confidence interval; CRC, colorectal cancer; RD, rate difference.

### 3.4 Sensitivity Analysis

The findings were generally robust across sensitivity analyses. After excluding 140 early‐onset CRC cases diagnosed within 6 months of an endoscopic examination, 248 early‐onset CRC cases remained in sensitivity Analysis 1. The age‐specific associations were similar to those in the primary analysis, with overall heterogeneity observed for both genetic risk (*p* = 0.001) and lifestyle (*p* = 0.047) (Table S11). No statistically significant additive interaction between genetic risk and lifestyle was observed for early‐onset CRC (Table S12). In sensitivity Analysis 2, where the cutoff age for early‐onset CRC was set at 50 years and the time axis was alternatively divided into four age groups (< 50, 50–59, 60–69, and ≥ 70 years), the HRs (95% CIs) for high genetic risk in relation to CRC diagnosed before age 50 reached 5.03 (2.55–9.90), with evidence of heterogeneity across age intervals for genetic risk (*p* = 0.001) (Table S13). In sensitivity Analysis 3, after excluding events that occurred within the first year of follow‐up to minimize potential reverse causation, the results remained stable (Table S14).

## 4 Discussion

In this large prospective cohort study, we evaluated the associations of genetic risk and lifestyle with CRC across different ages at onset and examined their additive interaction for early‐ and late‐onset CRC. For genetic risk, stronger relative associations were observed at earlier onset ages, with the clearest difference between CRC occurring before age 55 years and CRC occurring at age 75 years or older. This heterogeneity was primarily driven by the high versus low genetic risk contrast. Lifestyle associations also varied across onset‐age intervals. The heterogeneity was mainly attributable to the unfavorable versus favorable lifestyle contrast, particularly when comparing CRC occurring before age 55 years with CRC occurring at ages 55–64 and 65–74 years. In addition, a positive additive interaction between genetic risk and an unfavorable lifestyle was observed for late‐onset CRC, whereas no statistically significant additive interaction was detected for early‐onset CRC. These findings suggest that both the relative associations and the joint effects of genetic susceptibility and lifestyle with CRC vary across the life course.

To place our findings in the context of the existing literature, we conducted a structured PubMed search of studies investigating genetic susceptibility, lifestyle or environmental factors, and CRC according to age at onset. The full search strategy is provided in the Methods in Data S1, and the identified studies and their key differences from the present study are summarized in Table S15. However, much of this evidence has focused on overall CRC, early‐onset CRC alone, or individual environmental exposures. The present study extends this literature by evaluating genetic and comprehensive lifestyle associations across multiple onset‐age intervals, formally testing heterogeneity across age groups, and examining additive genetic and lifestyle interaction for early‐ and late‐onset CRC.

Inheritance accounts for approximately 25% of CRC cases diagnosed before age 50 and up to 33% of those diagnosed before age 35 [3], suggesting that genetic factors play a more prominent role in the development of early‐onset CRC. The younger the age at onset, the greater the contribution of hereditary predisposition, underscoring the importance of genetic testing and family history assessment in young adults. This finding is consistent with our results, indicating that genetic predisposition is more strongly associated with earlier onset CRC than with later onset CRC. Consistent with this pattern, we observed stronger relative associations between genetic risk and CRC at younger onset ages, whereas the association gradually attenuated with increasing age. However, the formal pairwise heterogeneity analysis indicated that the clearest difference was between CRC occurring before age 55 years and CRC occurring at age 75 years or older. In addition, although the association between high genetic risk and CRC diagnosed before age 50 was particularly strong in the sensitivity analysis, this estimate should be interpreted cautiously because of the limited number of cases in this age group.

Lifestyle‐related associations with CRC also varied according to onset age, although the pattern was less consistent than that observed for genetic risk. Compared with a favorable lifestyle, an unfavorable baseline lifestyle showed little association with CRC occurring before age 55 years, whereas stronger associations were observed for CRC occurring at ages 55–64 and 65–74 years, followed by a more modest association at age 75 years or older. A similar non‐monotonic pattern was observed when the healthy lifestyle score was analyzed continuously. These findings suggest that the association between lifestyle and CRC may vary across the life course rather than increase or decrease uniformly with age. The observed heterogeneity was mainly attributable to the unfavorable versus favorable lifestyle contrast, particularly when CRC occurring before age 55 years was compared with CRC occurring at ages 55–64 and 65–74 years.

Archambault et al. [27] previously evaluated the joint contribution of genetic and environmental susceptibility to early‐onset CRC using a 141‐variant PRS and a 16‐factor environmental risk score. Their study showed that both genetic and environmental risk contributed to early‐onset CRC and suggested possible positive interaction. Consistent with their findings, we observed a strong association between genetic susceptibility and CRC occurring at younger ages. However, we did not detect a statistically significant additive interaction between genetic risk and lifestyle for early‐onset CRC, whereas positive additive interactions were observed for late‐onset CRC. This difference may reflect differences in study design. Archambault et al. pooled data from 13 cohort and case–control studies across three international consortia, and in the case–control components, environmental risk factors were assessed retrospectively after cancer diagnosis. As acknowledged by the authors, these data may therefore have been susceptible to recall bias and may not fully capture the etiologically relevant exposure period for CRC development, particularly for exposures earlier in life. In contrast, our study used a prospective cohort design in which lifestyle factors were assessed before incident CRC diagnosis, thereby reducing the potential for recall bias and establishing a clearer temporal relationship between exposure and disease occurrence. In addition, our findings should be considered complementary to those of Archambault et al., extending their work by examining genetic and lifestyle associations across multiple ages at CRC onset and directly comparing additive interaction between early‐ and late‐onset CRC.

Our study has several strengths. First, lifestyle factors were assessed before incident CRC diagnosis, reducing the potential for recall bias and establishing temporal ordering between exposure assessment and disease occurrence. Second, the use of multiple onset‐age intervals, formal heterogeneity tests, continuous age‐varying analyses, and additive interaction measures provided complementary perspectives on age‐related variation in genetic and lifestyle associations. Finally, we conducted sensitivity analyses to further confirm the robustness of our findings.

Our study also has several limitations. First, the cohort mainly consisted of white participants, which limits the generalizability of the findings to other ethnic groups. Future studies across populations of different ancestries are warranted. Second, the selection biases cannot be completely ruled out due to participants in UK Biobank tending to be healthier than the general population. Third, the associations of genetic and lifestyle factors with CRC risk may vary according to tumor molecular characteristics, particularly microsatellite instability (MSI) status. However, tumor molecular data, including MSI status, were not available in this study. Fourth, lifestyle factors were assessed only at baseline and were not repeatedly measured during follow‐up. Therefore, our findings reflect associations with baseline lifestyle rather than the effects of lifestyle change. Finally, despite the large overall sample size, the number of early‐onset CRC cases remained relatively limited, resulting in imprecise estimates for some stratified and interaction analyses. Future studies with larger numbers of early‐onset CRC cases are needed to validate and extend these findings.

In conclusion, the associations of genetic susceptibility and baseline lifestyle with CRC risk varied across age at onset. Positive additive interaction between genetic risk and an unfavorable lifestyle was observed for late‐onset CRC, whereas no statistically significant additive interaction was detected for early‐onset CRC. These findings provide further evidence that the associations of genetic susceptibility and lifestyle with CRC are age‐dependent and may inform future research on age‐specific risk stratification and prevention.

## Author Contributions

**Wen Sun:** conceptualization, methodology, software, formal analysis, visualization, writing – original draft. **Di Liu:** conceptualization, validation, writing – review and editing, software, methodology. **Jingyang Liu:** writing – review and editing, validation. **Dongxue Wang:** writing – review and editing. **Xiao Wang:** writing – review and editing, methodology. **Deqiang Zheng:** writing – review and editing, supervision, data curation. **Jianguang Ji:** conceptualization, writing – review and editing, supervision, data curation.

## Funding

This work was supported by the start‐up grant from the University of Macau (UMDF‐TISF/2025/001/FHS, MYRG‐GRG2026‐00040‐FMD), a faculty PI allocated fund (FHS‐CC‐046‐001‐2024), Macao Science and Technology Development Fund (0111/2025/RIB2), and MAS Cancer to Jianguang Ji.

## Ethics Statement

The UK Biobank data were obtained with approval from the North West Multicenter Research Ethics Committee (MREC) (REC reference: 21/NW/0157). All participants provided informed consent in accordance with UK Biobank procedures. This research was conducted using resources from the UK Biobank under approved application number 95259.

## Consent

The authors have nothing to report.

## Conflicts of Interest

The authors declare no conflicts of interest.

## Supporting information


**Figure S1:** Flowchart of study enrollment.
**Figure S2:** Age‐specific cumulative incidence of colorectal cancer according to genetic risk and lifestyle categories.
**Figure S3:** Age‐varying associations of the polygenic risk score (PRS) and healthy lifestyle score with colorectal cancer (CRC).

**Data S1:** Supplementary methods.

**Table S1:** Healthy lifestyle score components.
**Table S2:** Characteristics of participants at baseline according to genetic risk groups.
**Table S3:** Baseline characteristics of participants according to colorectal cancer status and age at diagnosis.
**Table S4:** Cumulative incidence of colorectal cancer (CRC) according to genetic risk and lifestyle category.
**Table S5:** Category‐specific heterogeneity across all onset‐age intervals.
**Table S6:** Pairwise heterogeneity in genetic risk associations across onset‐age intervals.
**Table S7:** Pairwise heterogeneity in lifestyle associations across onset‐age intervals.
**Table S8:** Associations of genetic risk (per standard deviation) and lifestyles (per one‐factor) with CRC across onset‐age groups.
**Table S9:** Associations of lifestyle factors with early‐onset of colorectal cancer (CRC).
**Table S10:** Associations of lifestyle factors with late‐onset of colorectal cancer (CRC).
**Table S11:** Sensitivity analysis of the associations of genetic risk and lifestyle with colorectal cancer across age‐at‐onset groups after excluding early‐onset CRC cases diagnosed within 6 months of endoscopic examination.
**Table S12:** Sensitivity analysis for relative excess risks due to interactions between genetic risk and lifestyles on early‐onset colorectal cancer (CRC) after excluding early‐onset CRC cases diagnosed within 6 months of endoscopic examination.
**Table S13:** Sensitivity analysis for associations of genetic risk and lifestyles with different age onsets of colorectal cancer (CRC).
**Table S14:** Sensitivity analysis for associations of genetic risk and lifestyles with different age onsets of colorectal cancer (CRC) by excluding events occurring within the first year of follow‐up.
**Table S15:** Summary of studies investigating the risk of PRS and lifestyle associated with colorectal cancer.

## Data Availability

The data that support the findings of this study are available from UK Biobank. Restrictions apply to the availability of these data, which were used under license for this study. Data are available from https://www.ukbiobank.ac.uk/ with the permission of UK Biobank.
