# Supplementary material for: Genetic Risk, Lifestyle, and Colorectal Cancer Across Age at Onset: A Prospective UK Biobank Study
Source: Cancer Med. 2026 Oct 29;15(11):e72451. doi: 10.1002/cam4.72451 (PMC13720344; doi:10.1002/cam4.72451)
Supplement: Supplementary file 1 [file CAM4-15-e72451-s001.docx]

**Fig S1. Flowchart of study enrollment.**

CRC, colorectal cancer; PRS, polygenic risk score.

**Fig S2. Age-specific cumulative incidence of colorectal cancer according to genetic risk and lifestyle categories.**

(A) Cumulative incidence of colorectal cancer (CRC) across attained age according to genetic risk categories (low, intermediate, and high). (B) Cumulative incidence of CRC across attained age according to lifestyle categories (favorable, intermediate, and unfavorable). Non-CRC death was treated as a competing event. Solid lines indicate cumulative incidence estimates, and shaded areas represent 95% confidence intervals.

**Fig S3. Age-varying associations of the polygenic risk score (PRS) and healthy lifestyle score with colorectal cancer (CRC).**

(A) Hazard ratios (HRs) for CRC per 1-standard deviation increase in PRS;

(B) HRs for CRC per one-factor decrease in healthy lifestyle score.

Cox proportional hazards models were used to estimate the HRs and 95% confidence intervals adjusted for sex, ethnicity, Townsend deprivation index, and family history of CRC. The gray ribbons represent 95% confidence intervals and their centers represent the hazard ratios.
