# Supplementary material for: Genetic Risk, Lifestyle, and Colorectal Cancer Across Age at Onset: A Prospective UK Biobank Study
Source: Cancer Med. 2026 Oct 29;15(11):e72451. doi: 10.1002/cam4.72451 (PMC13720344; doi:10.1002/cam4.72451)
Supplement: Supplementary file 2 [file CAM4-15-e72451-s002.docx]

**Supplementary Methods**

A structured literature search was conducted in PubMed to identify studies examining genetic susceptibility, lifestyle or environmental risk factors, and colorectal cancer (CRC) according to age at onset. The search combined terms related to CRC, age at onset, genetic susceptibility, and lifestyle or environmental exposures. The following search strategy was used:

((((((((((Colorectal Neoplasms[Title/Abstract]) OR (colorectal cancer[Title/Abstract])) OR (colorectal cancers[Title/Abstract])) OR (colorectal neoplasm[Title/Abstract])) OR (colorectal neoplasms[Title/Abstract])) OR (colorectal carcinoma[Title/Abstract])) OR (colorectal carcinomas[Title/Abstract])) OR (colon cancer[Title/Abstract])) OR (colon cancers[Title/Abstract])) OR (rectal cancer[Title/Abstract])) OR (rectal cancers[Title/Abstract])

AND

(((((((((((((((((((early-onset[Title/Abstract]) OR (early onset[Title/Abstract])) OR (early-onset colorectal cancer[Title/Abstract])) OR (early onset colorectal cancer[Title/Abstract])) OR (EOCRC[Title/Abstract])) OR (young-onset[Title/Abstract])) OR (young onset[Title/Abstract])) OR (younger-onset[Title/Abstract])) OR (younger onset[Title/Abstract])) OR (late-onset[Title/Abstract])) OR (late onset[Title/Abstract])) OR (later-onset[Title/Abstract])) OR (later onset[Title/Abstract])) OR (LOCRC[Title/Abstract])) OR (age at onset[Title/Abstract])) OR (age-at-onset[Title/Abstract])) OR (age of onset[Title/Abstract])) OR (onset age[Title/Abstract])) OR (age at diagnosis[Title/Abstract])) OR (age-at-diagnosis[Title/Abstract])

AND

(((((((((((polygenic risk score[Title/Abstract]) OR (polygenic risk scores[Title/Abstract])) OR (genetic risk score[Title/Abstract])) OR (genetic risk scores[Title/Abstract])) OR (PRS[Title/Abstract])) OR (polygenic susceptibility[Title/Abstract])) OR (genetic susceptibility[Title/Abstract])) OR (genetic predisposition[Title/Abstract])) OR (genetic risk[Title/Abstract])) OR (genome-wide association study[Title/Abstract])) OR (genome-wide association studies[Title/Abstract])) OR (GWAS[Title/Abstract])

AND

(((((((((((((((((((((((lifestyle[Title/Abstract]) OR (lifestyles[Title/Abstract])) OR (lifestyle factor[Title/Abstract])) OR (lifestyle factors[Title/Abstract])) OR (healthy lifestyle[Title/Abstract])) OR (healthy lifestyles[Title/Abstract])) OR (environmental risk[Title/Abstract])) OR (environmental risk factor[Title/Abstract])) OR (environmental risk factors[Title/Abstract])) OR (modifiable risk factor[Title/Abstract])) OR (modifiable risk factors[Title/Abstract])) OR (diet[Title/Abstract])) OR (dietary[Title/Abstract])) OR (smoking[Title/Abstract])) OR (tobacco[Title/Abstract])) OR (alcohol[Title/Abstract])) OR (physical activity[Title/Abstract])) OR (sedentary[Title/Abstract])) OR (obesity[Title/Abstract])) OR (body mass index[Title/Abstract])) OR (BMI[Title/Abstract])) OR (waist-to-hip ratio[Title/Abstract])) OR (red meat[Title/Abstract])) OR (processed meat[Title/Abstract])
