# Supplementary material for: Genetic Risk, Lifestyle, and Colorectal Cancer Across Age at Onset: A Prospective UK Biobank Study
Source: Cancer Med. 2026 Oct 29;15(11):e72451. doi: 10.1002/cam4.72451 (PMC13720344; doi:10.1002/cam4.72451)
Supplement: Supplementary file 3 [file CAM4-15-e72451-s003.docx]

**Supplementary table 1.** Healthy lifestyle score components.

| **Characters** | **Detailed information** | **Score** |
| --- | --- | --- |
| BMI (kg/m^2^) | ≥18.5 and <25  <18.5 or ≥25 | 1  0 |
| Smoking | Never  Previous  Current | 1  0  0 |
| Waist-to-hip ratio | Men: <0.90  Women: <0.85  Men:>=0.90  Women: >=0.85 | 1  1  0  0 |
| Physical activity 10+ min (days/week) | Moderate 6-7 and vigorous 3-5  vigorous 6-7  Other | 1  1  0 |
| Sedentary time^a^ (hours/day) | <3  ≥3 | 1  0 |
| Red meat and processed meat intake^b^ | <4 times per week  >=4 times per week | 1  0 |
| Fruits and vegetables intake^c^ | >5 servings/day  ≤5 serving/day | 1  0 |
| Drinking status | Never  Previous  Current | 1  0  0 |
| Total Score Range |  | 0-8 |

^a^Sedentary time was calculated based on the sum of the time watching television and using computer.

^b^For red and processed meat, we summed the frequencies for beef, pork, lamb/mutton, and processed meat, using the following coding: “Never” = 0, “Less than once a week” = 0.5, “Once a week” = 1, “2-4 times a week” = 3, “5-6 times a week” = 5.5 and “Once or more daily” = 7.

^c^For fruit, participants were asked to enter the number of pieces of fresh fruit and dried fruit. one piece of fresh fruit, and two ‘pieces’ of dried fruit were counted as a serving. For vegetables, participants were asked to enter the number of heaped tablespoons of cooked vegetables and salad/raw vegetables eaten per day. Two heaped tablespoons of vegetables were counted as a serving.

**Supplementary table 2.** Characteristics of participants at baseline according to genetic risk groups.

| **Characteristics** | **Genetic risk groups** | | |
| --- | --- | --- | --- |
|  | **Low**  **(n= 101,364)** | **Intermediate**  **(n= 202,726)** | **High**  **(n= 101,363)** |
| **Age at baseline, median (IQR)** | 57 (49-63) | 57 (49-63) | 57 (49-63) |
| **Follow-up time, median (IQR)** | 13.64 (12.92-14.27) | 13.65 (12.92-14.28) | 13.66 (12.91-14.29) |
| **Incident CRC, N (%)** | 925 (0.91) | 3,113 (1.54) | 2,582 (2.55) |
| **Age group of onset CRC,**  **N (%)** |  |  |  |
| <55 | 45 (0.04) | 172 (0.08) | 170 (0.17) |
| 55-64 | 210 (0.21) | 774 (0.38) | 711 (0.70) |
| 65-74 | 457 (0.45) | 1,579 (0.78) | 1,281 (1.26) |
| >=75 | 213 (0.21) | 588 (0.29) | 420 (0.41) |
| **Sex, N (%)** |  |  |  |
| Female | 53,778 (53.05) | 107,446 (53.00) | 53,553 (52.83) |
| Male | 47,586 (46.95) | 95,280 (47.00) | 47,810 (47.17) |
| **Ethnicity, N (%)** |  |  |  |
| White | 94,328 (93.06) | 192,974 (95.19) | 97,971 (96.65) |
| Other | 7,036 (6.94) | 9,752 (4.81) | 3,392 (3.35) |
| **Townsend deprivation index, mean (SD)** | -1.37 (3.06) | -1.40 (3.03) | -1.39 (3.04) |
| **Lifestyle scores^a^, N (%)** |  |  |  |
| Favourable | 35,382 (34.91) | 68,593 (33.84) | 33,258 (32.81) |
| Intermediate | 45,441 (44.83) | 91,627 (45.20) | 46,069 (45.45) |
| Unfavourable | 20,541 (20.26) | 42,506 (20.97) | 22,036 (21.74) |
| **Family history of CRC, N (%)** |  |  |  |
| Yes | 9,356 (9.23) | 22,190 (10.95) | 13,775 (13.59) |
| No | 92,008 (90.77) | 180,536 (89.05) | 87,588 (86.41) |

^a^Participants were classified into unfavorable (0 and 1 score), intermediate (2 and 3 score), and favorable (≥ 4 score) lifestyle subgroups based on lifestyle scores ranging from 0 to 8.

Abbreviations: CRC, colorectal cancer; IQR, interquartile range; SD, standard deviation.

**Supplementary table 3.** Baseline characteristics of participants according to colorectal cancer status and age at diagnosis.

| **Characteristics** | **No incident CRC (N=398,833)** | **Incident CRC** | | | |
| --- | --- | --- | --- | --- | --- |
|  |  | **<55 years**  **(N=387)** | **55-64 years (N=1,695)** | **65-74 years**  **(N=3,317)** | **≥75 years**  **(N=1,221)** |
| Age at baseline, median (IQR) | 57 (49-63) | 45 (43-49) | 54 (51-58) | 63 (60-65) | 67 (65-68) |
| Follow-up time, median (IQR) | 13.67 (12.95-14.29) | 4.15 (2.18-7.67) | 5.77 (2.73-9.31) | 7.25 (4.19-10.26) | 11.26 (9.58-12.59) |
| Person-years^a^ | 5,327,475 | 1,901 | 10,385 | 24,024 | 13,406 |
| Sex, N (%) |  |  |  |  |  |
| Female | 211,871 (53.12) | 204 (52.71) | 780 (46.02) | 1,382 (41.66) | 540 (44.23) |
| Male | 186,962 (46.88) | 183 (47.29) | 915 (53.98) | 1,935 (58.34) | 681 (55.77) |
| Ethnicity, N (%) |  |  |  |  |  |
| White | 378,835 (94.99) | 363 (93.80) | 1,624 (95.81) | 3,248 (97.92) | 1,203 (98.53) |
| Other | 19,998 (5.01) | 24 (6.20) | 71 (4.19) | 69 (2.08) | 18 (1.47) |
| Townsend deprivation index, mean (SD) | -1.39 (3.04) | -0.96 (3.19) | -1.45 (3.04) | -1.53 (3.03) | -1.68 (2.88) |
| Genetic risk groups^b^, N (%) |  |  |  |  |  |
| Low | 100,439 (25.18) | 45 (11.63) | 210 (12.39) | 457 (13.78) | 213 (17.44) |
| Intermediate | 199,613 (50.05) | 172 (44.44) | 774 (45.66) | 1,579 (47.60) | 588 (48.16) |
| High | 98,781 (24.77) | 170 (43.93) | 711 (41.95) | 1,281 (38.62) | 420 (34.40) |
| Lifestyle scores^c^, N (%) |  |  |  |  |  |
| Favorable | 135,533 (33.98) | 136 (35.14) | 462 (27.26) | 774 (23.33) | 328 (26.86) |
| Intermediate | 180,154 (45.17) | 176 (45.48) | 783 (46.19) | 1,508 (45.46) | 516 (42.26) |
| Unfavorable | 83,146 (20.85) | 75 (19.38) | 450 (26.55) | 1,035 (31.20) | 377 (30.88) |
| Family history of CRC, N (%) |  |  |  |  |  |
| Yes | 44,320 (11.11) | 57 (14.73) | 252 (14.87) | 498 (15.01) | 194 (15.89) |
| No | 354,513 (88.89) | 330 (85.27) | 1,443 (85.13) | 2,819 (84.99) | 1,027 (84.11) |

Abbreviations: CRC, colorectal cancer; IQR, interquartile range; SD, standard deviation;

^a^Person-years were calculated as the sum of individual follow-up times within each group. Follow-up time was calculated from the date of baseline assessment to the date of CRC diagnosis, death, loss to follow-up or the last censoring date.

^b^Genetic risk was categorized into low (bottom quartile), intermediate (2^nd^-3^rd^ quartile) and high (top quartile) according to quartile cut-off points of the PRS.

^c^Participants were classified into unfavorable (0 and 1 score), intermediate (2 and 3 score), and favorable (≥ 4 score) lifestyle subgroups based on lifestyle scores ranging from 0 to 8.

**Supplementary table 4.** Cumulative incidence of colorectal cancer (CRC) according to genetic risk and lifestyle category.

| **Subgroup** | **Case/Total** | **Cumulative Incident function, % (95%CI)** |
| --- | --- | --- |
| **Genetic risk** |  |  |
| Low | 925/101,364 | 2.61 (2.30-2.91) |
| Intermediate | 3,113/202,726 | 3.96 (3.68-4.24) |
| High | 2,582/101,363 | 6.72 (5.84-7.60) |
| **Lifestyle** |  |  |
| Favourable | 1,700/137,233 | 3.72 (3.35-4.08) |
| Intermediate | 2,983/183,137 | 4.11 (3.80-4.42) |
| Unfavourable | 1,937/85,083 | 5.40 (4.63-6.18) |

Abbreviations: CI, confidence interval.

**Supplementary table 5.** Category-specific heterogeneity across all onset-age intervals.

| **Risk factor** | **Comparison** | ***P* for heterogeneity** |
| --- | --- | --- |
| Genetic risk | Intermediate vs low genetic risk | 0.053 |
|  | High vs low genetic risk | **<0.001** |
| Lifestyle | Intermediate vs favorable lifestyle | **0.010** |
|  | Unfavorable vs favorable lifestyle | **0.019** |

**Supplementary table 6.** Pairwise heterogeneity in genetic risk associations across onset-age intervals.

| **Risk factor** | **Age-at-onset** | ***P* for heterogeneity** | ***P* for heterogeneity: intermediate vs low** | ***P* for heterogeneity: high vs low** |
| --- | --- | --- | --- | --- |
| Genetic risk | <55 | Reference | Reference | Reference |
|  | 55-64 | 0.750 | 0.834 | 0.536 |
|  | 65-74 | 0.142 | 0.575 | 0.096 |
|  | ≥75 | **0.002** | 0.083 | **0.001** |

**Supplementary table 7.** Pairwise heterogeneity in lifestyle associations across onset-age intervals.

| **Risk factor** | **Age-at-onset** | ***P* for heterogeneity** | ***P* for heterogeneity: intermediate vs favorable** | ***P* for heterogeneity: unfavorable vs favorable** |
| --- | --- | --- | --- | --- |
| Lifestyle | <55 | Reference | Reference | Reference |
|  | 55-64 | 0.142 | 0.252 | **0.046** |
|  | 65-74 | 0.057 | 0.247 | **0.015** |
|  | ≥75 years | 0.220 | 0.394 | 0.353 |

**Supplementary table 8**. Associations of genetic risk (per standard deviation) and lifestyles (per one-factor) with CRC across onset-age groups.

| **Age group of onset** | **Case** | **Case per PY (1,000)** | **Genetic risk (per standard deviation)** | | **Lifestyle (per one-factor)** | |
| --- | --- | --- | --- | --- | --- | --- |
|  |  |  | **HR (95%CI)** | ***P* for heterogeneity** | **HR (95%CI)** | ***P* for heterogeneity** |
| <55 | 387 | 0.32 | 1.54 (1.39-1.70) | <0.001 | 1.02 (0.96-1.09) | 0.023 |
| 55-64 | 1,695 | 0.89 | 1.58 (1.51-1.66) |  | 1.10 (1.07-1.14) |  |
| 65-74 | 3,317 | 1.68 | 1.47 (1.42-1.53) |  | 1.12 (1.10-1.15) |  |
| >=75 | 1,221 | 2.53 | 1.34 (1.27-1.42) |  | 1.07 (1.03-1.11) |  |

Abbreviations: CRC, colorectal cancer; PY, person-years; HR, hazard ratio; CI, confidence interval.

Adjust for sex, ethnicity, Townsend deprivation index, and family history.

**Supplementary table 9.** Associations of lifestyle factors with early-onset of colorectal cancer (CRC).

| **Lifestyle** | | **Low genetic risk** | **Intermediate genetic risk** | **High genetic risk** |
| --- | --- | --- | --- | --- |
|  |  | **HRs (95% CIs)** | **HRs (95% CIs)** | **HRs (95% CIs)** |
| BMI | 1 | Reference | Reference | Reference |
|  | 0 | 1.12 (0.57-2.19) | 0.79 (0.56-1.12) | 0.68 (0.48-0.95) |
| Smoking | 1 | Reference | Reference | Reference |
|  | 0 | 1.70 (0.94-3.09) | 0.83 (0.60-1.14) | 1.11 (0.82-1.51) |
| Alcohol | 1 | Reference | Reference | Reference |
|  | 0 | 1.80 (0.23-14.10) | 1.70 (0.61-4.74) | 1.80 (0.56-5.81) |
| WHR | 1 | Reference | Reference | Reference |
|  | 0 | 0.97 (0.49-1.93) | 1.37 (0.96-1.95) | 1.31 (0.92-1.88) |
| Physical activity | 1 | Reference | Reference | Reference |
|  | 0 | 1.64 (0.58-4.61) | 1.22 (0.77-1.93) | 1.10 (0.71-1.72) |
| Sedentary time | 1 | Reference | Reference | Reference |
|  | 0 | 1.02 (0.54-1.90) | 0.91 (0.67-1.25) | 1.01 (0.73-1.39) |
| Fruit and vegetables intake | 1 | Reference | Reference | Reference |
|  | 0 | 0.77 (0.41-1.41) | 0.89 (0.65-1.23) | 1.06 (0.76-1.47) |
| Meat intake | 1 | Reference | Reference | Reference |
|  | 0 | **2.09 (1.13-3.86)** | 1.33 (0.97-1.81) | 1.05 (0.76-1.44) |

The Cox model included BMI, smoking, alcohol, waist-to-hip ratio, physical activity, sedentary time, fruit and vegetables intake, meat intake, sex, ethnicity, Townsend deprivation index, and family history.

Abbreviations: CRC, colorectal cancer; HR, hazard ratio; CI, confidence interval.

**Supplementary table 10.** Associations of lifestyle factors with late-onset of colorectal cancer (CRC).

| **Lifestyle** | | **Low genetic risk** | **Intermediate genetic risk** | **High genetic risk** |
| --- | --- | --- | --- | --- |
|  |  | **HRs (95% CIs)** | **HRs (95% CIs)** | **HRs (95% CIs)** |
| BMI | 1 | Reference | Reference | Reference |
|  | 0 | 1.07 (0.91-1.26) | 1.00 (0.91-1.09) | 1.17 (1.06-1.30) |
| Smoking | 1 | Reference | Reference | Reference |
|  | 0 | **1.24 (1.09-1.43)** | **1.21 (1.13-1.31)** | **1.22 (1.13-1.33)** |
| Alcohol | 1 | Reference | Reference | Reference |
|  | 0 | 0.96 (0.68-1.34) | 1.07 (0.87-1.32) | 0.94 (0.75-1.17) |
| WHR | 1 | Reference | Reference | Reference |
|  | 0 | 1.15 (0.98-1.35) | **1.24 (1.13-1.35)** | **1.21 (1.10-1.34)** |
| Physical activity | 1 | Reference | Reference | Reference |
|  | 0 | 1.14 (0.94-1.40) | 1.07 (0.96-1.19) | 1.04 (0.93-1.17) |
| Sedentary time | 1 | Reference | Reference | Reference |
|  | 0 | 1.07 (0.91-1.25) | 1.04 (0.95-1.13) | 1.01 (0.92-1.11) |
| Fruit and vegetables intake | 1 | Reference | Reference | Reference |
|  | 0 | 1.03 (0.90-1.18) | 1.07 (0.99-1.15) | 1.00 (0.92-1.08) |
| Meat intake | 1 | Reference | Reference | Reference |
|  | 0 | 0.99 (0.86-1.13) | 1.08 (1.00-1.17) | 1.07 (0.98-1.16) |

The Cox model included BMI, smoking, alcohol, waist-to-hip ratio, physical activity, sedentary time, fruit and vegetables intake, meat intake, sex, ethnicity, Townsend deprivation index, and family history.

Abbreviations: CRC, colorectal cancer; HR, hazard ratio; CI, confidence interval.

**Supplementary table** **11.** Sensitivity analysis of the associations of genetic risk and lifestyle with colorectal cancer across age-at-onset groups after excluding early-onset CRC cases diagnosed within 6 months of endoscopic examination.

| **Age group of onset** | **Case** | **Case per PY (1,000)** | **Genetic risk, HR (95%CI)** | | | | **Lifestyle, HR (95%CI)** | | | |
| --- | --- | --- | --- | --- | --- | --- | --- | --- | --- | --- |
|  |  |  | **Low** | **Intermediate** | **High** | ***P* for heterogeneity** | **Favorable** | **Intermediate** | **Unfavorable** | ***P* for heterogeneity** |
| <55 | 248 | 0.21 | Reference | 1.89 (1.26-2.83) | 3.41 (2.27-5.13) | 0.001 | Reference | 1.16 (0.88-1.55) | 1.25 (0.88-1.77) | 0.047 |
| 55-64 | 1,695 | 0.89 | Reference | 1.84 (1.57-2.14) | 3.34 (2.86-3.89) |  | Reference | 1.25 (1.11-1.40) | 1.51 (1.32-1.72) |  |
| 65-74 | 3,317 | 1.68 | Reference | 1.72 (1.55-1.91) | 2.80 (2.51-3.11) |  | Reference | 1.24 (1.13-1.35) | 1.58 (1.44-1.74) |  |
| >=75 | 1,221 | 2.53 | Reference | 1.38 (1.18-1.62) | 2.01 (1.71-2.38) |  | Reference | 0.96 (0.83-1.10) | 1.28 (1.10-1.49) |  |

Adjust for sex, ethnicity, Townsend deprivation index, and family history.

Abbreviations: CRC, colorectal cancer; HR, hazard ratio; CI, confidence interval.

**Supplementary table 12.** Sensitivity analysis for relative excess risks due to interactions between genetic risk and lifestyles on early-onset colorectal cancer (CRC) after excluding early-onset CRC cases diagnosed within 6 months of endoscopic examination.

| **Outcome** | **Age group of onset** | **Intermediate genetic risk, RERI (95%CI)** | | **High genetic risk, RERI (95%CI)** | |
| --- | --- | --- | --- | --- | --- |
|  |  | **Intermediate lifestyle** | **Unfavorable lifestyle** | **Intermediate lifestyle** | **Unfavorable lifestyle** |
| Early-onset CRC | <55 | -0.94 (-6.49-0.63) | -0.53 (-5.55-2.63) | 1.00 (-2.52-4.76) | -0.65 (-8.40-3.42) |

Adjust for sex, ethnicity, Townsend deprivation index, and family history.

The 95% CIs were derived by drawing 1,000 bootstrap samples.

Abbreviations: CRC, colorectal cancer; RERI, relative excess risks due to interactions; CI, confidence interval.

**Supplementary table 13.** Sensitivity analysis for associations of genetic risk and lifestyles with different age onsets of colorectal cancer (CRC).

| **Age group of onset** | **Case** | **Case per PY (1,000)** | **Genetic risk, HR (95%CI)** | | | | **Lifestyle, HR (95%CI)** | | | |
| --- | --- | --- | --- | --- | --- | --- | --- | --- | --- | --- |
|  |  |  | **Low** | **Intermediate** | **High** | ***P* for heterogeneity** | **Favorable** | **Intermediate** | **Unfavorable** | ***P* for heterogeneity** |
| <50 | 111 | 0.22 | Reference | 2.49 (1.26-4.92) | 5.03 (2.55-9.90) | 0.001 | Reference | 1.10 (0.73-1.65) | 0.88 (0.50-1.56) | 0.140 |
| 50-59 | 888 | 0.57 | Reference | 1.56 (1.27-1.92) | 3.19 (2.60-3.92) |  | Reference | 1.14 (0.98-1.33) | 1.39 (1.16-1.67) |  |
| 60-69 | 2,627 | 1.22 | Reference | 1.88 (1.67-2.12) | 3.07 (2.71-3.47) |  | Reference | 1.28 (1.16-1.41) | 1.55 (1.40-1.73) |  |
| >=70 | 2,994 | 2.22 | Reference | 1.54 (1.39-1.71) | 2.42 (2.17-2.70) |  | Reference | 1.10 (1.00-1.20) | 1.44 (1.31-1.59) |  |

Adjust for sex, ethnicity, Townsend deprivation index, and family history.

Abbreviations: CRC, colorectal cancer; HR, hazard ratio; CI, confidence interval.

**Supplementary table 14.** Sensitivity analysis for associations of genetic risk and lifestyles with different age onsets of colorectal cancer (CRC) by excluding events occurring within the first year of follow-up.

| **Age group of onset** | **Case** | **Case per PY (1,000)** | **Genetic risk, HR (95%CI)** | | | | **Lifestyle, HR (95%CI)** | | | |
| --- | --- | --- | --- | --- | --- | --- | --- | --- | --- | --- |
|  |  |  | **Low** | **Intermediate** | **High** | ***P* for heterogeneity** | **Favorable** | **Intermediate** | **Unfavorable** | ***P* for heterogeneity** |
| <55 | 349 | 0.29 | Reference | 1.82 (1.29-2.57) | 3.79 (2.69-5.35) | <0.001 | Reference | 1.13 (0.90-1.44) | 1.09 (0.80-1.47) | 0.022 |
| 55-64 | 1,541 | 0.81 | Reference | 1.83 (1.56-2.15) | 3.36 (2.86-3.95) |  | Reference | 1.22 (1.08-1.38) | 1.46 (1.27-1.68) |  |
| 65-74 | 3,187 | 1.61 | Reference | 1.70 (1.53-1.89) | 2.78 (2.49-3.10) |  | Reference | 1.23 (1.12-1.34) | 1.56 (1.42-1.72) |  |
| >=75 | 1,221 | 2.53 | Reference | 1.38 (1.18-1.62) | 2.02 (1.71-2.38) |  | Reference | 0.96 (0.84-1.10) | 1.28 (1.11-1.49) |  |

Adjust for sex, ethnicity, Townsend deprivation index, and family history.

Abbreviations: CRC, colorectal cancer; HR, hazard ratio; CI, confidence interval.

**Supplementary table 15.** Summary of studies investigating the risk of PRS and lifestyle associated with colorectal cancer.

| **Author (Year)** | **Data source** | **Study design** | **Sample** | **Genetic factor** | **Lifestyle/environmental factor** | **Age-at-onset analysis** | **PRS-lifestyle interaction** | **Main distinction from current study** |
| --- | --- | --- | --- | --- | --- | --- | --- | --- |
| Alexi N Archambault et al. (2022) | CCFR, CORECT, GECCO | case-control study | 3,486 EOCRC cases and 3,890 controls | CRC PRS, 141 variants | Environmental risk score, 16 factors | EOCRC <50 only | Additive interaction assessed between PRS and environment | Did not compare whether PRS-environment joint effects vary across CRC onset ages |
| Alexi N Archambault et al. (2021) | CCFR, CORECT, GECCO | case-control study | 3,767 EOCRC cases and 4,049 controls; 23,437 LOCRC cases and 35,311 controls | - | Anthropometric, dietary, lifestyle and pharmacological risk factors | EOCRC <50 vs LOCRC ≥50 | - | Comprehensively compared traditional non-genetic risk factors between EOCRC and LOCRC, but did not integrate PRS or a composite lifestyle score. |
| Alexi N Archambault et al. (2020) | Discovery: CCFR, CORECT, GECCO; Replication: RPGEH | Pooled case-control analysis with independent cohort replication | Discovery: 12,197 participants <50 and 95,865 ≥50; Replication: 72,573 participants | CRC PRS, 95 variants | - | EOCRC <50 vs LOCRC ≥50 | - | Directly established a stronger PRS association with EOCRC than LOCRC, but did not evaluate a comprehensive lifestyle score or age-specific PRS-lifestyle additive interaction. |
| Xuechen Chen et al. (2022) | DACHS study | case-control study | 5,104 CRC cases and 4,131 controls | CRC PRS, 140 variants | Alcohol consumption | EOCRC <55 vs LOCRC≥55 | Multiplicative interaction assessed between alcohol consumption and PRS | Examined one lifestyle exposure and two onset categories rather than an overall lifestyle profile across multiple age intervals |
| Jungyoon Choi et al. (2021) | UK Biobank | cohort study | 346,297 participants and 2,066 incident CRC | CRC PRS, 95 variants | Healthy lifestyle score, 8 factors | Overall CRC | Additive and multiplicative interactions between PRS and lifestyle | Did not investigate whether associations/interactions vary according to CRC age at onset |
| Mei Yang et al. (2023) | UK Biobank | case-control study | 2,585 CRC cases and 9,362 controls | CRC PRS, 140 variants | Several risk factors included | EOCRC <50 vs LOCRC ≥50 | - | Compared PRS associations in younger and older CRC using separate case-control models, but did not model attained-age-varying effects or joint comprehensive lifestyle effects. |
| Tung Hoang et al. (2023) | UK Biobank | cohort study | 374,004 participants and 4,686 incident CRC | CRC PRS, 98 variants | Dietary scores | Overall CRC | Multiplicative interaction assessed between PRS and dietary intake | Focused on dietary intake rather than an overall lifestyle profile and did not compare effects across CRC onset ages |
| Junyi Xin et al. (2023) | Multi-ancestry PRS development; UK Biobank used for prospective validation | cohort study | UKB: 355,543 participants and 2,621 incident CRC | Genome-wide PRS | healthy lifestyle scores, 8 factors | Overall CRC | Multiplicative interaction assessed between PRS and lifestyle | Focused on PRS development/validation and whether favorable lifestyle attenuates overall CRC risk, rather than age-dependent interaction patterns |
| Fu-Lan Hu et al. (2025) | UK Biobank | cohort study | 177,441 participants and 2,016 incident CRC | CRC PRS, 197 SNPs | EAT-Lancet Diet Index | Overall CRC | Additive interaction assessed between PRS and ELD-I | Demonstrated additive PRS-diet interaction for overall CRC, but did not compare effects across CRC onset ages and evaluate whether interaction differs across age at CRC onset |
| Sunny Sandhu et al. (2025) | UK Biobank | case-control study | 86 EOCRC cases and 430 controls; 5,108 LOCRC cases and 25,540 controls | UK Biobank standard polygenic risk score for CRC | Multiple lifestyle/metabolic factors | EOCRC <50 vs LOCRC ≥50 | - | Compared EOCRC and LOCRC risk factors, but used separate case-control models rather than prospective attained-age time-to-event modeling |
| Ruhina S Laskar et al. (2025) | EPIC, NOWAC and UK Biobank | cohort study | 1,068,703 participants and 14,859 incident CRC (1,369 EOCRC and 13,490 LOCRC) | - | 14 risk factors | EOCRC <55 vs LOCRC ≥55 | - | Strong prospective evidence for age-at-onset differences in lifestyle-related factors, but no CRC PRS or gene–lifestyle interaction analysis. |
| Fangyuan Jiang et al. (2023) | UK Biobank | Observational logistic-regression analysis | 113,256 participants; 165 EOCRC cases (143 prevalent, 22 incident); 719 early-onset adenomas | CRC PRS, 127 variants and FUT2 genotype | Long-term/recurrent antibiotic use during early life | EOCRC <50 only | Interaction assessed between long-term or recurrent antibiotic use and genetic factors | EOCRC-specific gene-environment study in UKB, but focuses on early-life antibiotic use and includes prevalent EOCRC; not a prospective age-spectrum comparison. |
| Yu Long et al. (2026) | UK Biobank | cohort study | About 430,000 participants | CRC PRS | Early-life tobacco exposure and age at smoking initiation | Overall CRC | Interaction assessed between tobacco and PRS | Addresses timing of tobacco exposure and genetic susceptibility rather than age-dependent PRS–overall lifestyle associations with CRC onset |
| Hannah Klinkhammer et al. (2026) | GC-FIC, HNR and UK Biobank | Cross-source comparative observational analysis | 5,739 individuals were included: 1839 GC-FIC probands, 3119 CRC-free HNR, and 781 were LOS-CRC cases from the UKBB | CRC PRS, 93 variants | - | EOS-CRC <50 vs LOS-CRC ≥75, plus familial/hereditary groups; not within one source population | - | Focused on PRS across clinically/genetically defined CRC groups; lifestyle was not evaluated |
| Haoxue Wang et al. (2023) | GECCO and UK Biobank | EOCRC-specific GWAS and PRS validation | GWAS: 17,789 CRC cases (1,490 EOCRC) and 19,951 controls; UKB PRS validation: 723 EOCRC and 24,427 controls | Established CRC loci plus EOCRC-specific susceptibility signals | - | EOCRC and age-at-diagnosis | - | Strengthens evidence for EOCRC-specific genetic susceptibility but does not evaluate lifestyle or age-varying joint effects in a cohort. |
| R.S. Laskar et al. (2024) | GECCO, CORECT, CCFR, and UK Biobank. | EOCRC-specific GWAS meta-analysis and two-sample MR | 6,176 EOCRC cases and 65,829 controls | EOCRC GWAS | 28 potentially modifiable risk factors | EOCRC <50 | - | Provides EOCRC-specific genetic and causal exposure evidence, but does not compare comprehensive PRS/lifestyle associations across attained ages. |
| E Wu et al. (2022) | UK Biobank | cohort study | 390,365 participants and 4,090 incident CRC | PRS, 59 variants | Healthy lifestyle score: 16 factors | Overall CRC | Additive and multiplicative Interaction between PRS and lifestyle | Highly relevant UKB PRS and healthy lifestyle study, but did not explore age-at-onset stratification. |
| Luo C et al. (2024) | UK Biobank and Kailuan cohort | cohort study and non-linear MR | UK Biobank: 374,568 participants, 4,656 CRC (357 EOCRC; 4,299 LOCRC); Kailuan: 172,809 participants, 936 CRC (112 EOCRC, 824 LOCRC) | PRS for fasting glucose, 70 variants | Blood glucose | <55 vs ≥55 | - | Shows that prospective UKB studies have formally compared an individual metabolic risk factor by CRC onset age; does not address comprehensive lifestyle and CRC PRS. |
| Present study | UK Biobank | cohort study | 405,453 participants and 6,620 incident CRC | UK Biobank CRC PRS derived from external GWAS | 8-factor comprehensive healthy lifestyle score | Four attained-age intervals: <55, 55-64, 65-74, ≥75; continuous age-varying analysis; | Additive interaction assessed between PRS and lifestyle according to age at onset | Integrates multiple attained-age intervals, continuous age-varying associations, age-specific additive interaction, and absolute-scale estimates. |

Abbreviations: CRC, colorectal cancer; PRS, polygenic risk score; EOCRC, early-onset colorectal cancer; LOCRC, late-onset colorectal cancer; HR, hazard ratio; CI, confidence interval; GWAS, genome-wide association study; MR, Mendelian randomization; SNP, single-nucleotide polymorphism.
